# From minding the gap to widening the gap: Paralympic athletes' experiences of wellbeing during the postponement of the Tokyo 2020 games

**DOI:** 10.3389/fspor.2022.921625

**Published:** 2022-08-26

**Authors:** Andrea Bundon, Lisa R. Trainor, Erica V. Bennett, Myriam I. Tremblay, Staci Mannella, Peter R. E. Crocker

**Affiliations:** ^1^School of Kinesiology, The University of British Columbia, Vancouver, BC, Canada; ^2^International Collaboration on Repair Discoveries (ICORD), The University of British Columbia, Vancouver, BC, Canada; ^3^Department of Counseling Psychology, Social Psychology, and Counseling, Ball State University, Muncie, IN, United States

**Keywords:** psychological wellbeing, sport, athlete, disability, pandemic, COVID-19

## Abstract

In March 2020, it was announced that the Tokyo Games would be postponed for one year due to the COVID-19 pandemic. While athletes commonly face challenges in sport such as injuries, the pandemic and rescheduling of the Games was an unexpected event that had serious potential to challenge the psychological wellbeing of athletes. Furthermore, it was an event that was simultaneously experienced by all athletes preparing for the Games. It provided a novel opportunity to explore how athletes navigated this challenging environment and the subsequent potential impact on their psychological wellbeing. It also provided a unique opportunity to engage para-athletes and explore how they experienced the pandemic and postponement. This manuscript draws on a larger qualitative study of 21 Canadian athletes (14 Olympic and seven Paralympic) who were on target to compete at the 2020 Games when the postponement was announced. For this manuscript, we focus on the accounts of seven Paralympic hopefuls and their experiences of adjusting to the postponement, while attending to the unique social identities of athletes with disabilities. Adopting a constructionist lens, semi-structured interviews were conducted at two time points. Through reflexive thematic analysis, we developed three themes. “We are all in the same boat. . . or are we?” describes the Paralympic hopefuls experiences early in the pandemic and how they felt united by the Canadian response to withdraw from the Games. It then discusses how, over time, they started to understand athletes with disabilities were being inequitably impacted by the pandemic and related public health measures. “Maybe it means more to them than us” examines how their perceptions changed as they acknowledged that although all athletes were facing a disruption to their sport careers, the implications were not the same for all. “Vulnerability and the Paralympic athlete” addresses how Paralympic athletes engaged with societal narratives about risk, vulnerability and disability and what this meant for the Paralympic Movement's response to the pandemic. “Honestly, I've experienced it before” examines how the Paralympic hopefuls drew on past experiences of injury to navigate the pandemic and the protective impact on their psychological wellbeing. Findings shed light on how systemic ableism interacted with the pandemic to magnify feelings of inferiority and further marginalization but also how para-athletes drew on past experiences to navigate challenges to their psychological wellbeing.

## Introduction

Current and retired elite athletes are reporting high rates of wellbeing related concerns (Rice et al., 2016) and with this increased awareness, sport organizations and governments are recognizing that a duty of care toward the athletes who represent our nations is imperative (Reardon et al., 2019; Henriksen et al., 2020). Yet, historically the extant research in sport psychology has presented a homogenous view of elite athletes, failing to account for athletes' diverse and intersecting identities (gender, sexuality, race, class, disability and more) that shape their abilities and their agency to act in ways that support, protect and enhance their own psychological wellbeing (Sparkes, 2013). While it is acknowledged in psychological theorizing that wellbeing is shaped by the social context (Lazarus, 1999; Ryff and Singer, 2006), the role of intersecting identities in shaping experiences of privilege and marginalization are often ignored in research and programming; thereby rendering inequitable access to the pursuit of psychological wellbeing in elite athletes. This failure to consider how athletes are differentially situated to respond to various challenges is not only a gap in the research but reproduces social inequities and marginalizes certain athletes by obscuring their experiences and what they need to survive and thrive (Schinke et al., 2019; Bundon and Mannella, 2022). Athletes with disabilities participating in para-sport^1^ are amongst those whose experiences have largely been neglected in sport and wellbeing research, although there is a growing body of work in this area as outlined next.

### Athlete wellbeing

In recent years, there has been a shift from studying the presence of psychopathy (or “illbeing”) to exploring how individuals experience wellness, wellbeing and psychological growth (Deci and Ryan, 2008). This work, however, is balanced with an awareness that a holistic approach to wellbeing must take into account both those aspects that support individuals to thrive and those that limit the possibilities of them experiencing optimal wellbeing. In the context of athlete wellbeing, this means being attentive to the conditions in which sport is practiced and how these conditions can foster physical, social and psychological benefits (Fraser-Thomas et al., 2005; Quested and Duda, 2010; Giles et al., 2020) and simultaneously expose athletes to negative experiences making them more vulnerable to psychological disruptions (Hammond et al., 2013; Orlowski and Wicker, 2018; Wicker et al., 2020).

While there is lack of agreement on a universal definition, wellbeing is generally conceptualized as including hedonic and eudemonic elements (Keyes et al., 2002). The hedonic perspective is conceived of as having a high degree of positive affect, low degree of negative affect and generally a positive assessment of current satisfaction with one's life (Diener, 2000; Deci and Ryan, 2008). The eudemonic perspective refers to the ability to actualize one's potential and to live well and is used synonymously with the term psychological wellbeing (PWB) (Deci and Ryan, 2008). Whereas hedonic wellbeing speaks to experiencing happiness and a sense of satisfaction in the shorter term, eudemonic wellbeing is more closely associated with cultivating a sense of meaning and fulfillment over the course of one's lifetime (Huta and Ryan, 2010). It is the eudemonic perspective (or PWB) that informed our research, although we acknowledge that hedonism and eudaimonism are strongly related and simultaneously experienced while being distinct ways of conceptualizing wellbeing (Heintzelman, 2018).

### Para-athlete wellbeing

Compared to studies of Olympic athletes, research into Paralympic athletes' psychological and behavioral processes and the influences on their wellbeing is underdeveloped, although there has been increased attention paid to the topic in the last 10 years (Jefferies et al., 2012; Martin, 2017; Swartz et al., 2019). While several studies have sought to identify the psychosocial characteristics of Paralympic athletes and other athletes with disabilities performing at an elite level, this research has often focused on ‘measuring’ the mental health (or prevalence of mental illness) in Paralympic athletes (Swartz et al., 2019). Other work has compared their mental health to that of other populations including individuals with disabilities who do not participate in para-sport and Olympians (Macdougall et al., 2017; Olive et al., 2021). A growing body of work has also paid attention to the applied practice and the delivery of psychological skills training in para-sport contexts when working with Paralympic athletes (Guerrero et al., 2020). Though much of the literature points to how athletes with disabilities face the same challenges in sport as their able-bodied peers and benefit from similar supports, there is also an acknowledgment that there are aspects of para-sport that are unique and very little is known about how these might impact upon an athlete's PWB. Foremost amongst these are the transitions into and out of para-sport (Bundon et al., 2018; Bundon, 2021; Martin and Prokesova, 2021), the process known as “classification” whereby athletes are assessed for their eligibility to compete in para-sport and placed into sport classes based on impairment type and/or physical functioning (Guerrero et al., 2020; Campbell and Brown, 2021), and experiences of trauma related to sustaining a major injury and acquired impairment (Martin et al., 2011; Swartz et al., 2019). Only very recently have researchers started to take a more holistic approach to understanding the PWB of elite athletes with disabilities that considers their intersectional identities and the social and cultural contexts in which discourses of ability/disability are (re)produced. Included in this body of work is Swartz et al. (2019) who state that the “study of mental health issues among Paralympic athletes will be affected by how people with disabilities are viewed socially” (p. 739) and as such must engage with issues of discrimination, marginalization, negative stereotypes and cultural understandings of disabilities.

### Ableist structures and psycho-emotional disablism

Building on the above cited literature it becomes clear that studies of Paralympic athlete wellbeing need to be attentive to the conditions in which sport is practiced and specifically how disability is understood in these spaces. In the Canadian context, where our research took place, this means looking at the process of “integration.” Sport Canada adopted policies to officially integrate disability sport programs into the mainstream (i.e., non-disabled) sport system starting in the mid-1990's. Under these policies, the responsibility for delivering para-sport programs and creating pathways for para-athletes to train toward the Paralympic Games became the responsibility of national sport organizations such as Athletics Canada or Rowing Canada (to give two examples), whereas previously these were the responsibility of disability specific sport organizations such as the Canadian Blind Sports Association or the Canadian Wheelchair Sports Associations. However, despite government policies and funding structures that stipulate that Canada has one high performance sport system, in many instances Paralympic athletes still train and compete within systems that are “somewhere between assimilation and segregation” (Howe, 2007, p. 146).^2^ For example, organizations frequently hold separate training camps for their Olympic and Paralympic teams and have different coaches and staff assigned to each group. The parallel arrangement of the Olympic Games followed a few weeks later by the Paralympic Games is an example of how competitions reproduce segregation whereas the Commonwealth Games that include events for athletes with and without disabilities is one of the few examples of an integrated competition in elite sport (Quinn et al., 2020). While much has been written on integration in sport contexts including exploring attitudes toward disability amongst those directly involved in integrated events (see for example, Misener et al., 2015; Paradis et al., 2017; Quinn et al., 2020), managerial perspectives on integration policies and sport delivery (Kitchin and Howe, 2014; Hammond et al., 2021) and the experiences of para-athletes in integrated organizations (Howe, 2007), little research has considered how the implications of integration shape and constrain athlete wellbeing.

One of the few publications to address the issue of integration in ‘regard’ to wellbeing is Campbell and Brown (2021). Drawing on Brown's experiences as a British para-athlete and Campbell's work as a performance psychologist within the national sport system, they address how the very structures and systems in place to ‘support’ athlete wellbeing are themselves imbued with ableism. The concept of “ableism” comes from critical disability scholarship and is the term used to describe policies, practices, and social arrangements that are premised on assumptions that ‘work for’ or privilege a normative, able-bodied subject but that negatively and inequitably impact those with disabilities. Campbell and Brown's (2021) statement that the structures in place to support athlete wellbeing are ableist references how the elite sport system in many nations (including Great Britain and Canada) was created for able-bodied athletes and only later amended to include para-athletes. However, because these systems were not created with para-athletes in mind, they often fail to provide what para-athletes need to thrive. Examples of ableist structures include requiring national team athletes to move to a centralized training center without considering the accessibility of housing or public transit available or leaving blind athletes to rely on teammates to guide them in airports when traveling rather than paying for an assigned guide.

Campbell and Brown (2021) call for sport practitioners within these spaces (practitioners who almost exclusively do not have lived experience of disability) to proactively build their awareness of disability related topics including issues of disability rights and advocacy and to reflexively engage with the ableist assumptions that underpin their personal practice and that of their sport organizations. Additionally, they introduce the concept of ‘psycho-emotional disablism’ to discuss the impacts and implications of being repeatedly exposed to discriminatory attitudes and/or ableist assumptions. Psycho-emotional disablism comes from the social relational model of disability and the theorizing of Carol Thomas (2007). It refers to the way that the wellbeing of individuals is undermined through social encounters with others or societal structures that are disabling, discriminatory or oppressive (Smith and Bundon, 2017). Thus, the integration of para-athletes into mainstream sport organizations that were built with ableist assumptions can be understood as exposing para-athletes to situations where their needs are regularly not being met or where they face discrimination and this has the potential to undermine their PWB.

### COVID-19 and understanding athlete psychological wellbeing

Competing at the Olympic or Paralympic Games is often seen as the pinnacle of one's athletic career. Athletes invest significantly in preparing for the Games and plan their lives around the quadrennial (Wylleman et al., 2012; Debois et al., 2015; Ryan, 2015). In March 2020, it was announced that the Tokyo Olympic and Paralympic Games would be postponed for one year due to the COVID-19 pandemic. While prior research into the psychological wellbeing of athletes has identified “disruptions” as significant events in athletic careers that have the potential to seriously challenge the psychological wellbeing of athletes, the focus has largely been on injuries, deselection or other events that are individually experienced (McEwen et al., 2018; Trainor et al., 2020). The extant literature has also largely attended to psychological wellbeing at particular moments and in response to specific stressors, rather than taking a more holistic and longitudinal approach that considers wellbeing as something that athletes experience over time and in relation to both temporary situations and the more enduring context of their athletic careers. The COVID-19 pandemic and the subsequent postponement of the Games provided a novel opportunity to explore the psychological wellbeing of elite athletes in that it was an unexpected event that was simultaneously experienced by all athletes preparing for the Olympic and Paralympic Games and disrupted their sport careers for a substantial period of time. Researchers have recognized the specific challenge that COVID-19 and the postponement pose to athlete wellbeing *and* the opportunity presented to use this moment to further our understanding of how athletes navigate challenging times. Early in the pandemic, Stambulova et al. (2020) published a commentary on the “challenges and possibilities” the pandemic posed to Olympic and Paralympic athlete development. Håkansson et al. (2021) followed suit with an editorial raising concerns about prolonged psychological distress amongst athletes due to the interruption to their careers and long-term “job insecurity” given the uncertainty about how and when sport will “restart.” Håkansson et al. (2021) also called for a focus on athletes whose participation is already marginalized including female athletes, athletes with disabilities, and athletes from developing countries, who they postulated would be disproportionally and inequitably impacted by any disruption and have fewer resources to sustain them during challenging times. While there is a burgeoning body of empirical literature on the experiences of elite athletes during the pandemic, including Olympic and Paralympic hopefuls preparing for Tokyo 2020 (see Dehghansai et al., 2021; Kubosch et al., 2021; Pensgaard et al., 2021; Urbański et al., 2021), many gaps remain. The gaps include empirical accounts of what athletes were feeling, experiencing and doing during the pandemic, leaving researchers to question what this particular moment in time can teach us about the broader context in which athlete wellbeing is shaped and experienced.

The postponement of the Tokyo 2020 Games – a moment when Paralympic athletes who were training toward the Games experienced an unexpected disruption in their sport careers – provided a unique opportunity to explore their experiences of psychological wellbeing and the implications pertaining to their intersecting social identities. Qualitatively exploring how these athletes experienced the pandemic contributes to our understandings of the tensions in how athletes with disabilities negotiate their various identities, how they manage within an ableist sport system, and how these experiences engender feelings of inclusion, marginalization, loss, and resilience that in turn contribute to their perceptions of and experiences with their psychological wellbeing.

## Method

We situate this research within a constructionist paradigm that seeks to understand the meanings that individuals create and attribute to their experiences (Tamminen and Poucher, 2020), while also acknowledging that these meanings and the lived experiences referenced are shaped and mediated by language, culture, and history (Willig, 2013). Moreover, this epistemological position assumes a critical stance where it is understood that knowledge and power are interconnected and researching from this paradigm “places a specific emphasis on examining the role of society, culture and power relations that contribute to individuals' constructions of their experiences” (Tamminen and Poucher, 2020, p. 543). This positionality is congruent with a social relational model of disability that understands disability and impairment as manifested with the context of social arrangements that include the ongoing medicalization of disability, the hegemony of ableism, and the cultural narratives surrounding disability (Smith and Bundon, 2017; Campbell and Brown, 2021). As such, the social relational model provides an opportunity to explore how experiences of disablism that are socially and contextually situated and impairment effects that are biologically situated shape people's activities and, in this case, their engagement in sport. Working from a relativist and subjective epistemology that understands knowledge as created transactionally in conversations between researcher and participants (and between research team members), we conducted semi-structured interviews and a reflexive thematic analysis (Braun and Clarke, 2019) to construct the findings presented in this manuscript.

### Procedures

This paper is part of a larger study that sought to examine elite athletes' psychological and emotional experiences during the postponement of the Tokyo 2020 Games. When the announcement was first made in March 2020 that the Games would be delayed, the research team sought and received approval from the University's Behavioral Research Ethics Board to recruit and interview Olympic and Paralympic athletes and hopefuls. Twenty-one athletes (*N* = 21; 14 Olympic, seven Paralympic) were purposefully sampled (Patton, 2015). The eligibility criteria stipulated that we were recruiting Canadian athletes who had already qualified and been selected for the 2020 Games or were attempting to qualify when the announcement was made that the Games were postponed. Given the different qualification timelines used by the various national sports organizations, it meant that our sample included athletes who had met the standards to be selected to the team but the team had not officially been announced. It also included athletes who were members of teams that had secured their “spot” at the Games but the roster of athletes was not yet confirmed. We refer to these athletes throughout the manuscript as “Olympic and Paralympic hopefuls” to acknowledge that although they were on a trajectory to attend the Games, their selection was not confirmed. Athletes were invited to participate in two semi-structured interviews. The first interview was scheduled as soon as possible after the athletes confirmed their willingness to participate and these interviews were conducted between March 2020 and July 2020. In the first round of interviews, athletes were asked about their initial experiences of the COVID-19 pandemic and the Tokyo 2020 Games postponement. Questions addressed what they were thinking and feeling when they first heard that Canada would not send a team and later when the Games were officially postponed, how they were experiencing the pandemic including coping and adapting to the interruption, and questions about how they perceived the pandemic and postponement to impact their wellbeing. Although the time lapse between recruiting the first participant in March and the last participant in July meant that those interviewed later were a bit more “distant” from the announcement of the postponement, during this timeframe there was still considerable uncertainty about if the Games would go ahead and what that would look like if they did.

We stopped recruiting new participants around the end of July and started scheduling second interviews because we heard from participants and from our own professional networks that competitions and selection events for Tokyo were starting to resume signaling to us that we were entering a different phase of the pandemic. The second round of interviews took place between October 2020 and February 2021 and each participant was interviewed again ~5 months after we first spoke with them. The intent of the second round of interviewing, and the decision to allow for a gap of several months between interviews, was to examine athletes' experiences over time including how they were coping and adapting to the postponement and if and how they perceived and experienced their psychological wellbeing to have changed over time. The time between interviews also allowed the research team to transcribe, start preliminary analysis of the data and discuss what would be important to include in the second interview. The second interview guide was tailored to be unique to the individual and follow up on items previously discussed but also included many standard questions including: how they were feeling about the pandemic and postponement at that moment, what challenges they had faced in the past 5 months, if/how they had perceived their wellbeing to have changed, and how they were feeling about the upcoming Games (and their preparation, attendance, etc.). Copies of both interview guides have been provided as Supplemental materials.

The interviews were conducted by the first, second and third author, all of whom have experience and expertise in qualitative research methodologies and qualitative interviewing. All interviews were conducted over Zoom in accordance with the university's directives at the time which stipulated that, where possible, researchers should employ methods that minimized in-person contact. The use of Zoom also facilitated interviews with athletes who were geographically dispersed across Canada. The interviews were (with the athletes' permission) audio-recorded and transcribed verbatim by members of the research team.

### Sample

Our final sample included 21 participants (14 Olympic hopefuls, seven Paralympic hopefuls). The seven Paralympic hopefuls included five women and two men and the athletes competed in para-athletics, para-cycling, para-triathlon, sitting volleyball, and wheelchair basketball. They ranged in age from 21 to 38 (M_age_ = 29.57) and had competed internationally in their current sport for 3–12 years. Three athletes had previously competed at one or more Paralympic Games and four were hoping to make Tokyo their debut Games.

### Data analysis

The interviews with the para-athletes lasted between 57 and 97 min for a total of 17.37 interview hours (9.75 h for first interviews, 7.62 h for the second interviews; total for interviews with all 21 athletes was 20.05 h with a M_time_ = 79.23 min). Data were analyzed through a reflexive thematic analysis (Braun and Clarke, 2019) and a constructionist lens. Cognisant that there are many approaches to thematic analysis, we position this work as a ‘reflexive’ thematic analysis (RTA) meaning that our process was not linear and cannot be described as a series of steps (Trainor and Bundon, 2021). Rather, we embarked in a process of engagement with the data both during and after the interviews were completed. During the interviewing, we held regular team meetings to discuss what we were hearing from athletes, had conversations about what to include and probe in the second round of interviews, and to make note of developing ideas. After interviews were transcribed, the first three authors independently (re)read the transcripts while making analytic annotations, and then added to the notes made by the other two. We shared our observations with the other members of the research team who made suggestions regarding literatures to explore and acted as “critical friends,” as we discussed, debated and challenged each other's interpretations. We also had ongoing discussions about our understandings of the data and reflected upon how our own positionalities, personal and professional experiences, and research interests were informing our analysis separately and combined. For example, there are many ways in which our research team is diverse: one has lived experience of disability, two have competed in the Paralympic Games (one as an athlete, another as a support person), three have worked with athletes as mental skills consultants, and the team includes graduate students and researchers at all career stages (early, mid and late). Team members also have different research specialities (e.g., PWB, stress and coping, cultural sport narratives, critical disability theories) and disciplinary training (e.g., sociology, psychology). In other ways, we might be considered a homogenous group: all have some experience of competing in sport at the university, national or international level. We all study or work at a Canadian research-intensive university and all identify as white, English speaking and cisgendered. These positionalities and experiences meant that we each brought a different lens to the work and approached the data from a unique perspective. In our conversations, we explored where our observations had converged and/or diverged and the implications therein. We moved from analytic notes (i.e., Author 2 made a note stating ‘silver of hope with postponement’, Author 3 tagged same comment with ‘Pride in Canada – it was the right thing to do’), to codes (i.e., relief it is postponement and not cancellation), to clusters of codes (i.e., overwhelmed with emotions), and eventually the development of themes. During this process we considered the data set as a whole (i.e., interviews with all 21 athletes). However, consistent with our epistemological position that understands experiences as shaped by social and cultural contexts, we also discussed how the interactions with the athletes and the accounts of their experiences were shaped by their social positionalities and identities (based on gender, age, ethnicity, disability and more). It was this line of questioning that prompted us to consider the interviews with the seven Paralympic athletes ‘on their own’ so that we could more robustly explore themes pertaining to disability, impairment, and ableism. It is important to note that our aim in this secondary analysis was not to compare the experiences of the Paralympic athletes to those of the Olympic athletes – an approach that we believe only serves to reproduce the marginalization of athletes with disability by suggesting that their experiences can only be understood through comparison to able-bodied athletes – but rather to work from a disability-centric position that acknowledges and affirms disabled identities. Thus, throughout this manuscript there are a few instances where the interviews with Olympic hopefuls are referenced but only when they serve to elaborate on the themes developed in discussion with the Paralympic hopefuls. Furthermore, it is important to note that working from this perspective means that our analysis is never done. Although we have arrived at a point where we have a better understanding of the phenomena we set out to study, we acknowledge that this analysis is contextually and temporally situated in addition to being co-produced and interpretive (Trainor and Bundon, 2021).

## Findings

In this section, three interrelated themes are presented that capture and describe the seven Paralympic hopefuls' experiences when the Games were first postponed and in the months that followed. In these themes, the participants navigated various social identities as well as discourses of solidarity, marginalization, internalized inferiority, and vulnerability. We discuss the implications of these findings drawing on the concept of psycho-emotional disablism and how experiences of marginalization undermine one's ability to thrive. The final theme addresses how past experiences prepared them to navigate this ‘unprecedented’ time.

### We are all in the same boat… or are we?

On March 22nd 2020, Canada became the first nation to announce that they would not be sending athletes to Tokyo if the Games were held as scheduled. This decision was made two days before the joint announcement by the International Olympic Committee (IOC), the International Paralympic Committee (IPC) and local organizing committee that the Games would be postponed until Summer 2021. Later, in an interview, Tricia Smith, Canadian Olympic Committee President explained that “we ha[d] to make this decision as global citizens—-for to control this pandemic is bigger than sport” (Dichter, 2020). Smith's comments on global citizenship and the need for the sport community to “do the right thing” resonated with the Canadian athletes we interviewed as reported elsewhere (Bennett et al., 2022). In those early days, the athletes experienced complex emotions including anger and sadness but also understanding and pride that the Canadians were prioritizing health and safety (Bennett et al., 2022). However, for the Paralympic athletes in our study, the announcement had another layer of complexity. For them, it was an opportunity to understand themselves as part of “Team Canada.” They experienced feelings of inclusion; they were part of a group of elite Canadian athletes who were simultaneously going through the same thing.

Kate: The members of Team Canada whether they are Olympians or Paralympians everybody found out the news at the exact same time. So you were a part of this like huge community. Whereas, things before it was always just like [my sport], vs. now it is just like we are all Team Canada so we were all sticking through this together. I found it actually kind of heartwarming […] The pandemic came on so quick for so many people that I know everybody is going through the exact same thing. […] like all of Team Canada went through the same thing. So it was cool to see all of Team Canada reach out to one another. […] We are all Team Canada, we are all representing the maple leaf.

This experience of feeling part of a singular Team Canada and equal to Olympians was reinforced by the coordinated announcements by the Canadian Olympic Committee (COC) and Canadian Paralympic Committee (CPC) that did not differentiate between Olympic and Paralympic athletes but communicated the notion that all athletes were “in the same boat” (Hannah) and that the Canadian team would stay home, stay safe, and train for 2021.

However, as the pandemic continued, cracks appeared in the narrative that everyone was in the same boat. Specifically, a few athletes encountered incidences where they perceived that although Olympic and Paralympic athletes were facing the same public health measures that limited access to training facilities and resulted in the cancellation of training camps and competitions^3^ – the implications of these measures were not the same for all. For example, one para-triathlete explained that while *all* athletes had limited (or constantly changing) access to swimming pools, this put *her* at a particular disadvantage because of her impairment.

Morgan: I can't swim, so that's the biggest thing. With my disability [a lower limb impairment], I try not to run too much. Swimming is really the way we increase volume. […] The volume was really up but it did kind of — I did get a bit of a knee injury because I wasn't swimming.

She went on to describe how she had to take time off training because she was doing so much running because it was the only type of training that she could consistently access during this time. Another participant expressed frustration that the postponement meant he would need to do yet another fundraiser because the financial support provided by his national federation was not enough and he was limited in how much income he could earn before the government clawed back his disability-related benefits. Although he appreciated the support he received from his community, he attributed the need to constantly fundraise to the ongoing marginalization of Paralympic athletes and their achievements. He stated that an Olympic athlete who had won the same number of medals as he had would not have such a difficult time getting funding, reinforcing para-sport as “second-class.”

Justin: I still have to do a fundraiser every year basically to end up covering all my yearly expenses because I'm living on my little income that I get from the insurance company and I am finding that if I want to cover all of my expenses for training in a year, I still have to do a fundraiser because I don't have sponsorships and stuff like that. So this is something that puts a lot of weight on me through the years. I am just fed up with putting on fundraisers every year. I know that with two medals at the Games – if I was on the Olympic side or if we would get as much for disability [sport] I wouldn't have to fight for it.

Additionally, athletes also described that because there are fewer athletes involved in para-sport and not as much depth in the fields, they typically need to travel to train with teammates or have any sort of competition. This meant that during 2020, while some able-bodied athletes were still able to meet up with teammates and participate in local training camps or competitions organized in compliance with local public health restrictions, para-athletes were still largely on their own or training with a single teammate. One participant also commented that many of the alternatives that were being developed to enable athletes to engage with others in online training groups or virtual competitions were not accessible to athletes with disabilities or designed with para-sport in mind.

Justin: [Speaking about the ongoing uncertainty about when he will next compete]. I am following the development of the able-bodied side and the ‘E’ world championships^4^ are coming up. I follow that but I mean I am not participating in those kinds of things because I know that there is so much discrepancies between measurement in the bike or the hand bikes.

While participants did not explicitly state that they perceived the public health restrictions, the resumption of some able-bodied events, or the investment in competitive options for able-bodied athletes in ‘virtual’ formats to be discriminatory, these examples do illustrate an awareness on the part of participants that athletes were no longer all ‘in the same boat’ and that para-sport and para-athletes were not being prioritized.

Other participants stated that they were seeing signs of able-bodied sport starting back up, but not para-sport.

Hannah: [Responding to a question about when she expected her university to resume wheelchair sports]. I think they're talking about or they are going to bring back football and stand-up basketball but I think they're only doing like a few sports. So I don't think we would be playing at least for the first semester.

Thus, this theme demonstrates that despite initial feelings of being part of a unified Team Canada, as the pandemic progressed, the Paralympic hopefuls started to question whether or not all national team athletes were indeed having the same experiences. The topics they raised spoke to the difference between equality and equity. For example, while all athletes had to deal with restricted access to facilities and travel limitations (equal or same situation), the impact on para-athletes was greater (different or unequitable situation). However, with the exception of Justin who compared his financial situation to that of Olympic athletes with comparable past Games results, none of the participants described these situations as discriminatory. Rather, they described these as unfortunate but unavoidable consequences or “simply the way things are.”

### Vulnerability and the paralympic athlete

In addition to the awareness described above that Paralympians and Olympians were experiencing the pandemic differently, participants also spoke about how globally individuals with disabilities were differently impacted by the pandemic and the need for the Paralympic Movement to protect their ‘at risk’ members. For example, when asked about the decision to postpone the Games, two participants referenced the need to advocate for Paralympians on the basis that they may have additional health concerns and compromised immune responses and thus be more vulnerable to COVID-19 compared to those without disabilities. Ali felt confident that the decisions that the IPC was making were in the best interests of Paralympians and that the IPC was, in their discussions with other stakeholders, advocating for athletes with disabilities with the full knowledge that their athletes have health concerns that Olympic athletes do not. She then went on to describe some of the discussions that were occurring in Canada around the time of her second interview with regard to whether or not Olympic and Paralympic athletes preparing for Tokyo should have priority access to the very recently approved vaccines^5^:

Ali: I think it is great that the CPC is advocating for us [to get priority access to vaccines]. Advocating for us as Paralympians, know[ing] that we have disabilities and some of us are more vulnerable. So I think that is really great and reassuring and validating.

Notably, this same athlete was the only one of the seven Paralympic hopefuls interviewed to perceive herself to be “more vulnerable to COVID-19” compared to able-bodied others.

Ali: It is my first pandemic since acquiring my disability so I have not lived through a pandemic with a disability and so – I don't want to say that I have been immune compromised – but having a disability is different than being able-bodied. Before I lived through H1N1 and SARS and all of those other pandemics but I was able-bodied and healthy and so I didn't have as much concern for my wellbeing. […] I was more confident in being able to recover from any illness or have mild symptoms and not really be impacted. Whereas, now because I have a disability, my body reacts differently to fatigue and exertion and so in that way I feel like I would be more at risk because I am constantly having to battle my body for energy. […] It has definitely been harder mentally and emotionally. Way harder than I thought it would be.

One other Paralympic hopeful stated that she recognized the need to advocate for and protect other para-athletes who she perceived to be more at risk for serious harm from COVID-19 infections.

Claire: [Concerns about getting infected] didn't impact me as a Paralympic athlete over an Olympic athlete so it is hard for me to really say too much. But I mean I did recognize… You know, our coaching staff sent out emails obviously from the Paralympic Committee just discussing the fact that this is a lot harder on a lot of Paralympic athletes. You know with different immune systems and different disabilities it can cause like a lot more stress and strain.

The remaining five participants either did not reference the issue of increased vulnerability for people with disabilities or stated that it was not pertinent to them because their particular impairment did not increase their risk of becoming infected or the severity of the outcomes if they were to become ill.

### Honestly, I've experienced it before

In contrast to the theme of enhanced vulnerability to COVID-19, the third and final theme addresses how the Paralympic hopefuls may have been uniquely positioned to deal with the challenges posed by the pandemic and the postponement. As stated in the introduction, there has been a shift away from thinking about wellbeing as something one either has or does not have to more holistic approaches that consider the extent to which one is able to experience wellbeing, considerations for achieving ‘optimal wellbeing’ and situations that thwart wellbeing. Included in this work is a growing awareness of how prior challenges – including injuries and trauma – can provide opportunities for growth that facilitate one's ability to navigate future challenges (Wadey and Everard, 2020). The accounts of the Paralympic hopefuls provided many illustrations of exactly this and particularly how those with acquired impairments drew parallels between their experiences during the pandemic and the injury that led to their impairment.

Ali described her experience of losing her leg as a period of time in which her wellbeing was “low”:

Ali: I would say when I first lost my leg, I felt a lack of balance. It is a huge adjustment going through trauma so that, yeah. That went on for quite a while […] Like everything is changing. Every aspect of your life is changing. And your disability is going to impact most parts of your life so it is like everything is uncertain except for maybe like your family or your friends. But even your relationships with your friends do change because unfortunately your ability changes and the things that you do change. So, you have less opportunity to hang out with the people you would normally see.

She then drew parallels between this experience and living through the pandemic and specifically the uncertainty she felt about the future and the sense that everything was changing. When first injured, she experienced changes to her activities because of the injury; now she was experiencing changes to her activities because of public health restrictions that limited gatherings and closed facilities. Both situations impacted her relationships with her friends because it changed what they could do together and where they could meet. However, this time, Ali was able to foresee the challenge to her wellbeing and intentionally took measures to maintain relationships with her friends.

Ali: That is something I was really on top of during the pandemic is like scheduling in walks with my friends like once or twice a week. My couple of friends that I have that like to walk and so I would make sure that I am every week booking a walk with a few of them. Not in a group but like one on one and then rotating.

Although Ali described the pandemic as something that was “hard” and was having a toll of her mentally and emotionally, she also felt that it was not “as bad as it could have been” because of the steps she took.

For another participant, the closures of sports facilities and cancellations of training camps and competitions caused her to reflect upon how she might eventually cope with sport retirement – and she compared it to her experience of “losing sport” before and how that experience had challenged her psychological wellbeing.

Hannah: There is going to be a day when I'm not going to be playing – or playing at a high level. […] That's huge. Yeah, I think it's something I definitely need to work on because one day I'm not going to be competing at a high level and if I don't have that balance and all of the sudden I lose this massive part of my life – it's going to be a struggle. Like I've got – honestly I've experienced it before – like I got injured. Stand-up basketball was my life and when I got injured it became very real that I'm not going to get to play stand-up anymore. I did not take it well. I really didn't handle it well at all so I know exactly what that looks like.

Thus, although the Paralympic hopefuls in this study understood the COVID-19 pandemic to challenge their psychological wellbeing in the moment, they also had examples of how they had experienced moments of low or less than optimal psychological wellbeing in the past. More specifically, several participants had the prior opportunity to experience life without sport or when their sport participation was significantly disrupted. This recognition that they had navigated uncertain times and/or had “lost” sport before only to find their way back gave some participants the confidence that they could navigate the current situation.

Additionally, three of the seven Paralympic hopefuls were employed outside of sport and three were in full time postsecondary studies meaning that six of the seven had other life areas in which they were significantly invested. Although school and workplaces were also disrupted by the pandemic, participants spoke about how they had been able to focus on their employment or studies during this time and how this had provided them with some structure to their days and helped to mitigate the disruption caused by the postponement of Tokyo.

One participant also referenced the different “pathways” to the Paralympic Games compared to the Olympic Games:

Zak: I think there are a lot of Olympic athletes who have a lot more invested, right? They have been doing, a lot of them have been doing this sort of stuff since they were 10 years old. And then for Paralympic athletes, a lot of them you have been introduced to it a bit later in life. Like after an injury or something like that. So the pathways are different, the lifetime investment is a little different. […] Yeah, I think some of the Olympic guys, [the postponement] was a little harder for them for sure. Just because of their level of investment I can definitely understand is a bit higher more often than not.

This same athlete went on to state that he was not sure that he would “miss” the hype if the Games proceeded with a reduced program because he was used to Paralympic sport not getting a lot of attention and there are always fewer fans for disability sport events anyways. Zak's comments can be read as an example of internalized disablism in that he appears to accept the notion that the Paralympic Games and Paralympians are less “valued” than Olympians. It also speaks to how athletes might have been differently impacted by the pandemic because of different “investments” in their sporting careers and athletic identities. The Paralympic hopefuls in the study were more likely than the Olympic hopefuls to be working or studying in addition to their sport careers (at least at the start of the pandemic, some Olympic hopefuls did later use the postponement as an opportunity to pursue educational or entrepreneurial pursuits as discussed in Bennett et al., 2022). Many of the Paralympic hopefuls had also started their sport careers a little later in life (after an injury) or had grown up doing a different sport before realizing they were eligible for or could be competitive at the Paralympic Games. In short, Paralympic hopefuls were more likely to have spent time prior to the pandemic investing in other areas of their lives outside of sport and it appears this might have had a protective effect on their psychological wellbeing when sport was disrupted.

## Discussion

The purpose of this paper was to examine how athletes with disabilities preparing for Tokyo 2020 experienced the pandemic, and explore how these experiences were shaped by the ableist structures of the sport environment in which they operate and the subsequent impact of these experiences on their psychological wellbeing. In the days that immediately followed the announcement that the Canadian delegation was pulling out of Tokyo unless the Games were postponed, the Paralympic hopefuls experienced complex emotions similar to those reported by their Olympic counterparts (Bennett et al., 2022). However, their accounts of that time also spoke of the pride they felt at being part of Team Canada and of sharing the experience with their “Olympic teammates.” This sentiment of being part of a singular Team Canada was unremarked upon by the Olympic hopefuls in the study but salient to the Paralympic hopefuls precisely because they do not always feel part of the same team.

In the first theme “*We are all in the same boat… or are we?”* the Paralympic hopefuls expressed how important it was to them to be seen as equal and part of the “same team,” using the announcement of the Canadian withdrawal from the Games to reinforce narratives of a Team Canada that included both Olympians and Paralympians. This desire to be affiliated with Olympians is tied to the “athletes first” narrative commonly reported in disability sport research whereby athletes with disabilities express a preference to be recognized for their athletic abilities and achievements (i.e., be seen as an “athlete first”), rather than for their identity as a person with a disability. While this discourse can be viewed as individually empowering, it has been widely critiqued by disability scholars for contributing to the ongoing marginalization of disabled people because it reinforces stereotypes that athletic identities are more valued that disabled identities (Braye, 2019). This theme is also an example of what has been termed the “Olympification” (Gérard, 2020) of Paralympic sport in that there is an underlying assumption that the Olympics/Olympians are the standard that the Paralympics/Paralympians should be aspiring to reproduce and thus an expression of internalized ableism. This can have detrimental implications during sport retirement because outside the “sport bubble” individuals with disabilities are still marginalized and discriminated against in broader society (Bundon et al., 2018; Martin and Prokesova, 2021). This resonates with what we observed in this study, where Paralympians had a moment where they felt included and saw themselves as on the same team but that experience was fleeting.

Indeed, by the time of the second interview, when the Paralympic hopefuls had a few months of living with the threat of COVID-19, several participants started to understand that there were discrepancies in how athletes were variously positioned to weather the pandemic. It is worth noting that this awareness of inequities was not limited to sport contexts and much has already been published on how the COVID-19 pandemic exposed and exacerbated existing fault lines in our societies. Individuals and communities who were already marginalized were also the most likely to experience negative and severe impacts from both the virus itself and the measures taken to mitigate the spread.^6^ Although no Paralympic hopefuls in this study explicitly stated that they believed the public health measures or the actions of their sports' organizations during the pandemic were discriminatory, they had examples of how their needs had not been prioritized in decisions pertaining to access to training facilities, opportunities to train and compete online, or restarting sport. For disability scholars and disability advocates, this finding will not be surprising but is merely another example of how hierarchies of ability and the hegemony of ableism mean that the needs of people with disabilities are an afterthought – when they are considered at all. Indeed, the unique contribution of our research is in documenting and providing empirical evidence for what others have already theorized would be the impact on athletes with disabilities. For example, Kitchin et al. (2022) built on the work of disability theorists to address how the pandemic would further inequities in sport unless intentional measures were taken by sport managers and policy makers to ensure access for those with disabilities and an equitable restart. The findings of our study, particularly the comment by one athlete, that she did not expect wheelchair basketball to start up again soon even though other university sports were resuming and the observation by another that there was little interest or investment in online/Esport competitions for para-athletes, confirm what Kitchin et al. (2022) observed globally. This is also consistent with previously published work, most notably Silva and Howe (2019) and referenced in Kitchin et al. (2022) that illustrates that discrimination in sport does not always occur because the individuals responsible for delivering sport are “anti-disabled,” but rather because they are working from a “pro-non-disabled” position.

In regard to understanding the impact of these pro-non-disabled decisions on the psychological wellbeing of the Paralympic hopefuls, we can draw on the concept of the internalization of ableism (Peers, 2012). As previously stated, none of the participants expressed that they felt discriminated against or perceived their lack of access to training and competition to be anything other than “expected.” Yet the themes contain many nuanced references to the supposed superiority of non-disabled sport. The internalization of attitudes and actions that devalue para-sport and athletes with disabilities can be understood as psycho-emotional disablism (Swartz et al., 2019) and damaging to the athletes' psychological wellbeing and ability to thrive by placing limits “on what one *can do* and *can become*” (Smith and Bundon, 2017, p. 22, italics in original).

The first theme also details the ongoing struggles of Paralympic athletes to financially support their sport careers and the additional stress and anxiety this entails. All of the Paralympic hopefuls in this study received a stipend from Sport Canada and their funding remained in place during the pandemic. However, as detailed by Justin, this stipend typically covers only some of the costs involved in being an elite athlete competing internationally and postponing the Games by a year meant he had to host an additional fundraiser to make it to Tokyo – an added stressor that he attributed to the lower status of Paralympic athletes. This issue of how the continued lack of material support for Paralympic athletes causes stress and anxiety for athletes and contributes to feelings of inferiority that have damaging implications for psychological wellbeing is one that warrants further research. We also see in this theme another link to how athletes with disabilities and para-sport are devalued based on material economic rhetoric. Justin references the Esport championships held for cycling on Zwift and the lack of opportunities for handcyclists in these virtual events. This resonates with statements made by disability advocates and reported by Kitchin et al. (2022) that technology can reduce some barriers related to accessibility, and thus many disability communities had extensive experience *prior to the pandemic* of using these platforms to connect. Indeed, prior research has pointed specifically to how Paralympic athletes were early adopters of digital tools and have effectively used technology to stay connected and provide practical and emotional support among networks of athletes who are geographically distant (Bundon and Hurd Clarke, 2015; Bundon, 2016). Yet *during the pandemic*, the knowledge and experience of these same communities was overlooked and disabled people were generally left behind in the rush to develop online and virtual platforms (i.e., online exercise classes).

In the second theme, Paralympic hopefuls discuss issues of risk and vulnerability in relation to COVID-19. First, it must be acknowledged that COVID-19 is a novel disease and in the early days of the pandemic very little was known about the virus and particularly who was most susceptible to contracting the virus and to experiencing severe outcomes if infected. Although we know a lot more two years into the pandemic, our understanding of COVID-19 is still evolving. What is clear, is that individuals with disabilities have been disproportionally affected by the disease and are more likely to experience severe illness and higher rates of mortality. For example, in England, a nation with some of the most centralized tracking of COVID-19 statistics, disabled people make up two-thirds of COVID-19 related deaths (Office of National Statistics, 2020). However, as stated in Penfold and Kitchin (2022), the public and policy discourses during the pandemic have frequently homogenized people with disabilities – often using euphemisms that reference to those with “underlying health conditions” (see also Sanyaolu et al., 2020) – and inappropriately positioned *all* people with disabilities as vulnerable. These discourses fail to acknowledge that being healthy and having a disability are not mutually exclusive states and might also undermine the psychological wellbeing of people with disabilities by labeling them as vulnerable and increasing anxiety. This labeling of particular groups as vulnerable has also been used to justify the exclusion of people with disabilities as sport restarts [i.e., suggesting that disabled sports fans do not return to live events even when the stadiums re-open to others (Penfold and Kitchin, 2022)].

In our findings, the Paralympic hopefuls largely distanced themselves from these discourses of risk and vulnerability. Only one spoke of how COVID-19 was her first time experiencing a pandemic as a person with a disability (the 2003 SARS and 2009 H1N1 outbreaks having occurred prior to her injury) and how she was acutely aware of being more vulnerable. None of the other participants expressed that they felt they were personally more vulnerable which speaks to the above statements that individuals with disabilities and their experiences during the pandemic have been inappropriately homogenized. Some of their statements can even been interpreted as reproducing stigmatizing and patronizing discourses as they refer to “others” – more disabled than themselves – who need protection. This theme makes the point that privilege and marginalization are not mutually exclusive – the Paralympic hopefuls in this study described themselves as both disadvantaged by certain pandemic restrictions and simultaneously more fortunate than others who were more vulnerable to disease.

It is in the final theme, “*Honestly, I've experienced it before”* that the athletes explicitly discuss their psychological wellbeing and the impact that the pandemic has had on it. As presented in the findings, some of the Paralympic hopefuls had acquired impairments and they referenced being injured and the time surrounding those injuries as a moment in their lives when their psychological wellbeing was low. This finding is not unique in that prior research has reported that injury is a stressful event that disrupts and challenges psychological wellbeing (Smith et al., 1990; Trainor et al., 2020) and there is a growing body of research into the role of trauma in the lives of Paralympic athletes and implications for post traumatic growth (Hammer et al., 2019; Brighton, 2020). More notable is how athletes made connections between this prior experience of being injured and their present-day assessments of their psychological wellbeing. The participants in this study unanimously acknowledged that the COVID-19 pandemic and the postponement of the Games was stressful and induced anxiety. Although they differed in their appraisals of their psychological wellbeing at the time of the interviews and in the months between (see Trainor et al., 2021), all participants felt their psychological wellbeing had been challenged during the pandemic and “less than optimal” for at least a part of this time. However, several of the participants expressed that having had those prior experiences with severe and life-altering injuries made them more resilient and prepared them to navigate the challenge of the pandemic. For some, this ability to cope was because of measures they had put in place years ago when first injured. This builds on other research finding that while injury can challenge psychological wellbeing it also affords athletes opportunities for growth; specifically athletes can emerge from injuries with more diversified identities, a greater appreciation for things they previously took for granted (such as their health and ability to engage in sport), and generally more resilient and prepared to navigate subsequent challenges to their wellbeing (Wadey et al., 2011, 2013; Roy-Davis et al., 2017; Trainor et al., 2020). Our study builds on this work by providing empirical evidence of how some athletes with disabilities “recognized” the threat to their wellbeing posed by COVID-19 and drew on past experiences to mitigate the negative impacts of the pandemic on their psychological wellbeing and/or to accept that their psychological wellbeing would be less than optimal for a time but that this too would pass. Finally, while we hesitate to draw conclusions based on the limited examples in our data, the athletes' comments regarding the “investment” of Paralympic athletes do raise some interesting questions regarding how pathways into Paralympic sport might impact upon an athlete's PWB. Future work could consider if and how starting an elite career later in life (after an injury or as new sports are added to the Paralympic program) might shape one's investment in the sport, the strength of one's athletic identity, and/or how one cope and adapts to challenging situations.

## Conclusion

The findings from this research detail the experiences of Paralympic hopefuls during the COVID-19 pandemic and how these experiences were shaped by their understandings of themselves as elite Canadian athletes and people with disabilities. Although all elite athletes preparing for the 2020 Olympic and Paralympic Games were faced with an unexpected and significant disruption to their athletic careers, this research demonstrates that not all athletes were in the same position to “weather the storm.” Paralympic hopefuls' experiences of the pandemic started with feelings of inclusion and they took pride in being of Team Canada. This shifted to realizations that their needs were not being prioritized during lockdowns or the resumption of sport. They also engaged with discourses that positioned them, their teammates and competitors as at risk and vulnerable to COVID-19 and in need of protection. All of these experiences can be understood to potentially impact their psychological wellbeing through psycho-social disablism that undermines one's ability to thrive when one is constantly exposed to discriminatory attitudes and behaviors and/or not provided with what one needs. Despite this, the Paralympic hopefuls drew on past experiences and specifically moments where their psychological wellbeing had been “low” to navigate present day circumstances. They recognized the pandemic as a similar moment where their wellbeing would once again be challenged and they drew on previously established supports and strategies.

In addition to providing an empirical account of Paralympic hopefuls' experiences during the COVID-19 pandemic, this research contributes to a growing body of work that considers the mental health and psychological wellbeing of elite athletes as shaped by social contexts and particularly intersecting identities and experiences of privilege and marginalization. In attending to the unique experiences of elite athletes with disabilities, it contributes to the ongoing task of making visible the ways that they engage in and with sport and laying a foundation for more equitable access to what they need to pursue psychological wellbeing.

## Data availability statement

The datasets presented in this article are not readily available because given the nature of these interviews it is not possible to provide anonymized data. Requests to access the datasets should be directed to andrea.bundon@ubc.ca.

## Ethics statement

The studies involving human participants were reviewed and approved by University of British Columbia—Behavioral Research Ethics Board. The patients/participants provided their written informed consent to participate in this study.

## Author contributions

AB, LT, and EB contributed to the conceptualization of the study, interviewing, data analysis/theme development, and writing (AB was the lead writer). MT and SM contributed to transcription, preparing sample description, and literature searches. PC contributed to conceptualization of the study, theme development, and provided feedback on the writing. All authors contributed to the article and more broadly to the research project described therein, and approved the submitted version.

## Funding

This research was funded by University of British Columbia Faculty of Education SSHRC Explore Grant and the School of Kinesiology Research Accelerator Fund.

## Conflict of interest

The authors declare that the research was conducted in the absence of any commercial or financial relationships that could be construed as a potential conflict of interest.

## Publisher's note

All claims expressed in this article are solely those of the authors and do not necessarily represent those of their affiliated organizations, or those of the publisher, the editors and the reviewers. Any product that may be evaluated in this article, or claim that may be made by its manufacturer, is not guaranteed or endorsed by the publisher.

## Footnotes

^1^Throughout the manuscript we use the terms ‘athletes with disabilities’ or ‘athlete with a disability’ as this is the convention within the Canadian sport system and the terminology most frequently used by the athletes themselves in the context of the interviews. We acknowledge that the debates that exist and that other individuals and communities adopt different terminology, most notably the use of the term ‘disabled athlete’ to reference that disability is socially constructed and individuals are ‘disabled’ by inequitable environments and discriminatory interactions. We use the term ‘para-sport’ to reference those sports that are on the Paralympic Games program and the focus of this research whilst acknowledging that athletes with disabilities participate and compete in a diverse array of sports both ‘segregated’ and with able-bodied athletes.

^2^Canada is not unique. Integration or ‘mainstreaming’ in sport became a priority for many Western nations around the same time (see Hammond and Jeanes, 2018).

^3^It is important to note that in Canada, public health measures are generally provincial jurisdiction. Athletes were located in various provinces across Canada, where COVID-related restrictions varied from full lockdowns (i.e. residents only permitted to leave home for essential services or if deemed to be an essential employee) to closures of some facilities (i.e. closure of gyms and fitness centres). That said, at the time of the first interviews, none of the athletes were training with teammates due to social distancing guidelines and all athletic competitions had been cancelled. Most athletes trained from home with equipment they had or borrowed from their sport organizations. At the time of the second interview, most provinces had in place policies that provided elite athletes registered with national training centres some exemptions to public health orders including access to their regular training facilities. However, this access was generally quite limited both in terms of the number of people that could be in attendance and the time allowed at the facility. Competitions or training camps were sometimes permitted but travel restrictions meant these events were often limited to athletes already living in the region.

^4^The Union Cycliste Internationale (UCI) which is the world governing body for cycling, introduced ‘Esports’ events in 2020 including the first world championships for online cycling in December 2020. Held on the platform Zwift, it included a men's and women's race on a virtual 50-kilometer course. Notably no event was held for handcyclists.

^5^As previously noted, the second round of interviews took place from October 2020 to February 2021. Canada approved the Pfizer-BioNTech COVID-19 vaccine for emergency use in early December 2020 and others shortly after. As with other public health measures, the administration of vaccines was provincial jurisdictions and each province had a different method of deciding which populations would be prioritized. However, at the time we were speaking with athletes, the vaccines were either not yet approved or were approved but only available to a small number of Canadians with most provinces deciding to prioritize front line healthcare workers and older adults living in care homes.

^6^The analogy of ‘being in the same storm but not in the same boat’ is one that was oft repeated during the pandemic as a way of describing how individuals and communities were differently positioned to ‘weather the storm’ based on intersecting lines of power, privilege and access to resources. We use the phrase because it was one repeated by participants and that aided us in conceptualizing this work and how athletes all experienced the same postponement of the Games but were differently impacted but we can hardly claim it as unique to our work. At the time of writing, a search of Google Scholar found over a dozen academic articles that contained some variation of this phrase in their titles.
