# Supplementary material for: From minding the gap to widening the gap: Paralympic athletes' experiences of wellbeing during the postponement of the Tokyo 2020 games
Source: Front Sports Act Living. 2022 Aug 26;4:921625. doi: 10.3389/fspor.2022.921625 (PMC9459162; doi:10.3389/fspor.2022.921625)
Supplement: Supplementary file 1 [file Data_Sheet_1.docx]

**Covid-19 and the Postponement of the 2020 Tokyo Games: Coping, Adaptation, and Psychological Wellbeing in Olympic and Paralympic Hopefuls**

**Interview Schedule**

*The following questions represent an overarching agenda for interviews with participants. The questions will be pursued flexibly and may be altered and added to over time as different themes are discussed by participants.*

**Interview #1**

**INTRODUCTION**

1. Can you tell me about your athletic background?
   1. What are some of your athletic highlights or most memorable moments?
   2. What are some challenges that you have experienced in sport?

*Coping and adaptation*

1. What has it been like to experience the Covid-19 pandemic?
   1. What areas of your life have been impacted by the pandemic?
   2. When did you start becoming aware of the pandemic and what did you think the implications would be?
      1. When did the pandemic become a concern to you?
2. Paralympians: What is it like to experience the pandemic while living with an impairment?
3. When did you start thinking the 2020 Tokyo Games might be postponed? What were you thinking and feeling?
   1. When you became aware of the uncertainty of the 2020 Tokyo Games, what were you thinking and feeling?
   2. How did you feel about Canada taking an early stance and deciding not to take a team to the Tokyo 2020 Games?
4. When and how did you learn that the 2020 Tokyo Games were postponed?
   1. What was it like to learn that the 2020 Tokyo Games were postponed?
   2. What thoughts and feelings came up for you when you heard about the postponement initially? Why?
   3. What were some of the things you did to manage this postponement initially?
5. What do you think of the 2020 Tokyo Games postponement now?
   1. How has your thinking and feelings about the postponement of the Games changed since the initial announcement, if at all?
   2. What are some of the things you are doing to manage this postponement now?
6. How has your athletic training and competitive schedule been impacted by the Covid-19 pandemic?
   1. Prior to the pandemic, what was it like to prepare for the Games?
      1. *Probe for any changes throughout the pandemic
7. Would you like to compete in the Tokyo 2021 Games?
   1. *Probe about selection and qualification for Games
   2. What factors do you anticipate causing you stress leading up to Tokyo 2021? (e.g. obstacles, challenges)
   3. How do you anticipate managing the stress you will experience leading up to Tokyo 2021?
   4. How might where you are residing benefit or hinder progression toward your athletic goals in this upcoming year?
   5. How do you feel about the possibility of the rescheduled 2021 being cancelled?
8. How are you being supported during this time, if at all? (e.g. probe for family, friends, teammates, coaches, sporting organizations)
   1. How has this form of support changed since Covid-19 and the postponement of the Games?
   2. What other support might you need during this time?
9. How has Covid-19 and the postponement of the Games impacted your financial situation, if at all?
10. What have you observed regarding how the Canadian Olympic/Paralympic movement has reacted to the postponement of the Games?
    1. How do you feel about what has been done in relation to the postponement of the Games?
    2. Have you participated in or engaged with any of these activities or discussions? If so, how?
       1. Have you been a part of any discussions or actions with networks at the international level? If so, can you elaborate?
    3. How has this involvement (if they have been involved) impacted how you make sense of and have dealt with the postponement of the Games?
    4. Have you observed any differences in how Paralympic athletes have responded that strikes you as different to how Olympic athletes have responded? Explain.
11. What are your future plans beyond the Olympics?
    1. How has the Covid-19 pandemic impacted these future plans, if at all?

*Well-being*

1. What are your top three concerns right now?
   1. How has your mental health and well-being been affected during this time?
   2. What have you done to support your mental health and well-being during this time?
2. In your opinion, what does optimal overall well-being look like for you?
   1. What does optimal overall well-being feel like to you?
   2. Can you provide some examples of what you might need around you to foster optimal overall well-being?
   3. What do you do to optimize your overall well-being?
   4. Can you provide an example of a time where you felt you ‘had’ optimal overall well-being? (why)
   5. Currently, amidst the pandemic, how do you compare to this ‘optimal’ overall well-being?
3. Conversely, what does a lack of overall well-being look like for you?
   1. What does a lack of overall well-being feel like for you?
   2. What might happen for one to get to a place where they lack overall well-being?
   3. If you were in this situation, what might you need or do to revive your overall well-being?
   4. Can you provide an example of a time where you felt you lacked overall well-being? (why, how to improve)
   5. Currently, amidst the pandemic, how do you compare to this ‘lack’ of overall well-being?
4. We’ve talked about this notion of well-being, having optimal overall well-being or lacking overall well-being. What would you say if I ask you what sport well-being means to you?
   1. How does overall well-being differ from sport PWB, if at all?
      1. What components might make up sport well-being?
      2. What kinds of things do you think help strengthen your sport well-being? (*probe for structures around the person)
      3. What kinds of things do you think hinder your sport well-being?
      4. Can you provide an example of a time in sport where you felt you ‘had’ optimal sport well-being? (why)
      5. Can you provide an example of a time in sport where you felt you ‘lacked’ optimal sport well-being? (why)
5. How might support from others (coaches, teammates, family) help or hinder your sport well-being?
   1. What does ideal support look like for you?
6. How might sport well-being change over the course of time?
7. How has COVID-19 impacted your sport well-being, if at all?
   1. What did your sport well-being look like when you anticipated the Games might be postponed?
   2. How about when the postponement was confirmed?
   3. How about over the last few months?
   4. What does your sport well-being look like now?
   5. What have you been doing to support your sport well-being during this time?
8. How has COVID-19 impacted your overall well-being, if at all?
   1. What did your overall well-being look like when you anticipated the Games might be postponed?
   2. How about when the postponement was confirmed?
   3. How about over the last few months?
   4. What does your overall well-being look like now?
   5. What have you been doing to support your overall well-being during this time?
9. To what extent do you think overall PWB and sport PWB are connected?

**WRAP UP**

1. How do you think the Covid-19 pandemic and postponement of the Games will change how athletes train and compete in the future, if at all?
2. Is there anything we have not discussed about your athletic experience, the postponement of the Tokyo Games, and Covid-19 that you think is important?

**Interview #2**

1. How have you been doing?
   1. What have the last 4/5 months been like for you?
   2. What has your everyday life looked like over the last 4/5 months?
      1. Has this changed over time? How and why?

**Probe for – What have you been thinking?*

*How have you been feeling?*

*What have you been doing?*

1. How do you feel about the Covid-19 pandemic currently?
   1. How has this changed over the last 4/5 months, if at all?
   2. How concerned are you about Covid-19?
2. Have you experienced any new challenges over the last 4/5 months?
   1. How have you dealt with these challenges?
   2. What challenges do you anticipate leading up to Tokyo 2021?

**(Probe for sport and other life domains)*

1. How do you feel about the postponement of the Games now?
   1. How has this changed since we last spoke, if at all?
   2. How are you feeling about Tokyo 2021 (do you think the Games will happen, why)? Would you (still) like to compete in the Tokyo 2021 Games?
2. How have training and competition been going?
   1. How has your training changed over the last 4/5 months, if at all?
   2. How has your competitive schedule changed over the last 4/5 months, if at all?
3. What is currently causing you stress, if anything?
   1. What do you anticipate being your main sources of stress leading up to Tokyo 2021?
4. How would you describe your wellbeing currently?
   1. How has your wellbeing changed over the last 4/5 months, if at all?
   2. How have you been supporting your wellbeing lately?
      1. How has this changed over the last 4/5 months, if at all?
5. Who has been supporting you over the last 4/5 months?
   1. How has it changed over the last 4/5 months, if at all?

**(who you are receiving it from, and how much you are receiving)*

- 1. How has your sporting organization been of support in the last 4/5 months?
  2. How adequate has this support been for you?
  3. What other support might you need?

1. What makes you feel fulfilled in life?
   1. What makes you feel fulfilled in sport?

**(probe for links between winning and feeling fulfilled)*

1. Is it possible to have optimal psychological wellbeing and be an elite athlete? Why or why not?
   1. How might success be tied to optimal psychological wellbeing, if at all?
      1. Do you need one to have the other?
      2. How might they be connected, if at all?
   2. How might failure impact psychological wellbeing, if at all?

*Probe for connection between PWB & performance

*Losing and winning & how this might impact PWB

1. Is there anything else you would like to add that we did not talk about today?

***For every athlete, also devise **specific questions** to follow up on based on first interview
